# Frailty as a predictor of mortality among patients with COVID-19: a systematic review and meta-analysis

**DOI:** 10.1186/s12877-021-02138-5

**Published:** 2021-03-17

**Authors:** Xiao-Ming Zhang, Jing Jiao, Jing Cao, Xiao-Peng Huo, Chen Zhu, Xin-Juan Wu, Xiao-Hua Xie

**Affiliations:** 1 Department of Nursing, Chinese Academy of Medical Sciences - Peking Union Medical College, Peking Union Medical College Hospital (Dongdan campus), Beijing, 100730 China; 2 Shenzhen Second People’s Hospital/the First Affiliated Hospital of Shenzhen University Health Science Center, Shenzhen, 518000 China

**Keywords:** Frailty, Mortality, COVID-19, Older adults, Meta-analysis

## Abstract

**Background:**

A large number of studies have explored the association between frailty and mortality among COVID-19 patients, with inconsistent results. The aim of this meta-analysis was to synthesize the evidence on this issue.

**Methods:**

Three databases, PubMed, Embase, and Cochrane Library, from inception to 20th January 2021 were searched for relevant literature. The Newcastle–Ottawa Scale (NOS) was used to assess quality bias, and STATA was employed to pool the effect size by a random effects model. Additionally, potential publication bias and sensitivity analyses were performed.

**Results:**

Fifteen studies were included, with a total of 23,944 COVID-19 patients, for quantitative analysis. Overall, the pooled prevalence of frailty was 51% (95% CI: 44–59%). Patients with frailty who were infected with COVID-19 had an increased risk of mortality compared to those without frailty, and the pooled hazard ratio (HR) and odds ratio (OR) were 1.99 (95% CI: 1.66–2.38) and 2.48 (95% CI: 1.78–3.46), respectively. In addition, subgroup analysis based on population showed that the pooled ORs for hospitalized patients in eight studies and nursing home residents in two studies were 2.62 (95% CI: 1.68–4.07) and 2.09 (95% CI: 1.40–3.11), respectively. Subgroup analysis using the frailty assessment tool indicated that this association still existed when using the clinical frailty scale (CFS) (assessed in 6 studies, pooled OR = 2.88, 95% CI: 1.52–5.45; assessed in 5 studies, pooled HR = 1.99, 95% CI: 1.66–2.38) and other frailty tools (assessed in 4 studies, pooled OR = 1.98, 95% CI: 1.81–2.16). In addition, these significant positive associations still existed in the subgroup analysis based on study design and geographic region.

**Conclusion:**

Our study indicates that frailty is an independent predictor of mortality among patients with COVID-19. Thus, frailty could be a prognostic factor for clinicians to stratify high-risk groups and remind doctors and nurses to perform early screening and corresponding interventions urgently needed to reduce mortality rates in patients infected by SARS-CoV-2.

**Supplementary Information:**

The online version contains supplementary material available at 10.1186/s12877-021-02138-5.

## Background

A global pandemic of coronavirus disease 2019 (COVID-19) was first reported in Wuhan city, China, in December 2019 [1]. The total number of confirmed cases was 96,877,399 worldwide on 23 January 2021 (https://www.worldometers.info/coronavirus/), with the highest mortality among older adults from different geographic regions, resulting in a huge burden for every sector of society, especially the global healthcare system. It has been reported that older adults living in community-dwelling or nursing homes are the most vulnerable group with the highest mortality rates for COVID-19 because of a variety of comorbidities and lower levels of immunologic function compared to younger adults [2]. Identifying the risk factors for predicting mortality among patients with COVID-19 is significant for clinicians.

Recently, many factors have been prognosticated for mortality, such as age [3], diabetes [4], hypertension, and obesity [5]. However, it has been reported that these somatic conditions cannot comprehensively predict worse outcomes for COVID-19 patients. Thus, new prognostic risk factors are required for identifying and stratifying patients.

Older adults are characterized by heterogeneity of health and vigor. Single aspects, such as chronological age and concurrent disease, cannot truly reflect overall health status. To solve this knowledge gap, frailty syndrome has been widely introduced in recent decades. Frailty is defined as a condition characterized by weakness, progressive declined physiologic function and diminished strength, leading to vulnerability and reduced resilience to stressors with an increased risk of adverse outcomes [6]. Frailty was confirmed to be a predictor of risk with worse outcomes, such as falls, mortality and lower quality of life in different populations [7]. Several studies have presented the association between frailty and morality in patients with COVID-19, the majority of which have shown a clear association between increasing frailty and worse outcomes [8–13]. Three small and underpowered studies showed no association [14–16]. Given recent articles exploring this association between frailty and mortality [17–23], we believe that there is an urgent need to summarize the evidence of this important issue. The objective of our study is to systematically review and quantify the results of the associations between frailty and mortality, which could provide evidence-based suggestions for clinicians.

## Methods

This meta-analysis followed the guidelines of the Preferred Reporting Items for Systematic Reviews and Meta-Analyses Statemen. We have registered our protocol in the PROSPERO database (CRD42021235666).

### Search strategy

Three databases - PubMed, Embase, and Cochrane Library - were independently searched by two authors (XPH, CZ) from database inception to 20th January 2021. We also used a combination of keywords and medical subject headings (MeSH). The search strategy was below frail* or frailty (MeSH) and (“COVID-19” OR “Coronavirus Infection” OR “Coronavirus Infection Disease 2019” OR Coronavirus*) and (mortality or death or survival). Additionally, we tried to find relevant studies from references and searched for gray studies using the Google search engine. The detailed search strategy for PubMed is shown in supplemental file 1.

### Inclusion and exclusion criteria

All observational studies describing the associations between frailty and mortality in patients with COVID-19 were included. We excluded article types such as comments, reviews, conferences, correspondence, editorials, letters to the editor and case reports. In addition, the study presented the effect size of the association by using frailty score as a continuous variable.

### Study selection process

Two authors blindly screened the literature with Endnote software (Clarivate Analytics,USA) after storing all of the relevant articles. The first step was to delete the duplications and then check the articles by title and abstract, finally identifying full texts that met the the of inclusion and exclusion criteria. When there was a disagreement, the third author participated in making a final decision.

### Data extraction

Two authors (XMZ and XHX) independently extracted the variables from the articles that were included, including basic characteristics (author, county, publication year, average age, prevalence of males/females, sample size, setting, and effect measures), study design, prevalence of frailty, frailty assessment scale, outcomes, effect size for the association with frailty, detailed variables of each adjustment model and mortality. The third author confirmed the final version of the date when there seemed to be any argument.

### Quality assessment

Two authors (XMZ and XHX) performed a quality bias assessment using the Newcastle–Ottawa Scale (NOS), which is widely applied for observational studies. The total score ranged from 0 to 9 points, and the higher the score, the higher the quality of the study. There are three categories for the level of quality: low (0–4), moderate (5–7) and high (> 7).

### Statistical analysis

The effect size (HR and OR) of the association between frailty and mortality was extracted by two independent authors (JJ and JC) with Microsoft Excel and was analyzed using STATA. The heterogeneity between the different studies was presented as *I*^*2*^ and was detected by Cochran’s Q test. The standard for the category of heterogeneity was defined as *I* ^2^ > 50% for significance and *I* ^2^ < 50% for insignificance. A random model was used to calculate and separately pool the effect size of HR and OR with a 95% CI between frailty and mortality because of the use of different populations, study designs, and various frailty assessment scales. In addition, subgroup analysis was also performed based on population, geographical region, different adjustment models and frailty assessment scales. Publication bias and sensitivity analysis were conducted by funnel plots.

### Search results and study characteristics

**Fig. 1:**
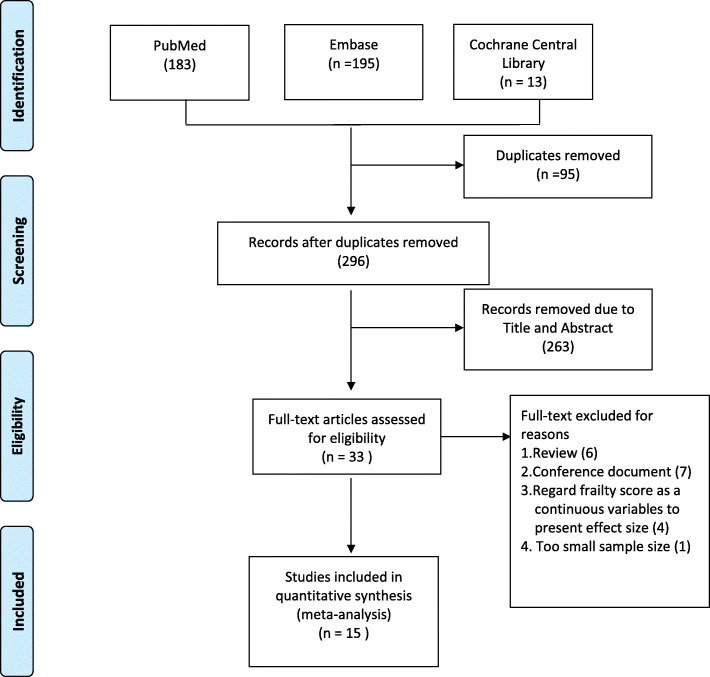
Research screening flowchart

We found 391 relevant articles from three databases, PubMed (183), Embase (195), and Cochrane Library (13). After using Endnote software to delete duplications, 296 articles remained. At this stage, two authors checked the titles and abstracts to identify closely relevant studies, with 33 assessed for eligibility. Consequently, after checking the full texts, 15 studies were included for quantitative analysis in terms of the predefined inclusion criteria. Detailed information for the reasons for exclusion is presented in Fig. 1.

Fifteen studies quantified the relationship between frailty and mortality that included 23,944 patients with COVID-19(shown in Table 1). Overall, a majority of the included studies focused on older adults. The study design in seven studies was prospective cohort studies [8, 12, 17–20, 22], while the others were all retrospective cohort studies [11, 13, 15, 16, 21, 23–25]. There were a variety of countries, ranging from the U.S. to European countries, with 2 in the USA [11, 24], five in the UK [8, 13, 15, 19, 22], 1 in Turkey [18],1 in France [16], 1 in Switzerland [23], 2 in Spain [21, 25], 2 in Sweden [12, 20], and 1 in the UK and Italy [17]. Of these, 13 study settings were in the hospital [8, 11–13, 15–20, 22, 23, 25] and two were in a nursing home [21, 24]. The prevalence of frailty ranged from 11.00 to 79%, and the pooled result was 51% (95% CI: 44–59%) (Supplemental Fig. 1). The majority of outcomes were in-hospital mortality, with three studies reporting 30-day mortality and one reporting 60-day mortality. The largest sample size was in Turkey [18], with 18,234 patients, and the smallest was in France [16], with 94 patients. Among all of the included studies, 11 studies used the clinical frailty scale (CFS) as an assessment tool for frailty [8, 12, 13, 15, 17, 19–23, 25], one used the hospital frailty risk score [18], one used the frailty index [24], one used the palliative performance scale [11] and one used the frail nondisabled questionnaire [16]. Detailed information of adjustment variables for each study was shown in Supplemental Table 1.

**Table 1:** Summary of Included Studies on frailty associated with Mortality among patients with Covid-19

Author	Design	County	Male%	Setting	Prevalence of frailty	Sample size	Age/years	Frailty Criteria	Effect measures	Outcome assessed
Kundi 2020 [18]	PCS	Turkey	46.6%)	Hospital	67.40%	18,234	74.1 (7.4)	HFRS	OR	In-hospital mortality
Hägg 2020 [12]	PCS	Sweden	48%	Hospital	44.10%	250	81.01 (8.56)	CFS	HR	in-hospital mortality
Aw 2020 [8]	PCS	UK	54%	Hospital	70.70%	677	81.1 (8.1)	CFS	HR	In-hospital mortality
Hewitt 2020 [17]	PCS	UK and Italy	58%	Hospital	51.25%	1564	74 (64–83)	CFS	HR	In-hospital mortality
Shi 2020 [24]	RCS	USA	44.10%	Nursing home	71.30%	139	85.0 (9.3)	Frailty index	OR	In-hospital mortality
Maguire 2020 [13]	RCS	UK	55.30%	Hospital	37.70%	224	older than 20 years	CFS	OR	30-day mortality
Cobos-Siles 2020 [25]	RCS	Spain	57%	Hospital	35.15%	128	84 (75, 89)	CFS	OR	In-hospital mortality
Steinmeyer 2020 [16]	RCS	France	45.00%	Hospital	11.00%	94	85.5 ± 7.5	FNDQ	OR	In-hospital mortality
Owen 2021 [15]	RCS	UK	54.00%	Hospital	53.30%	206	78.8 (8.3)	CFS	OR	In-hospital mortality
Fiorentino 2020 [11]	RCS	USA	NA	Hospital	64.10%	374	NA	Palliative Performance Scale	OR	In-hospital mortality
Chinnadurai 2020 [19]	PCS	UK	61.9%	Hospital	51.20%	215	74 (60–82)	CFS	OR	In-hospital mortality
Apea 2021 [22]	PCS	UK	69.70%	Hospital	51.90%	831	Aged 16 years or over	CFS	HR	30-day mortality
Tehrani 2021 [20]	PCS	Sweden	NA	Hospital	30.0%	143	Aged> 65 years	CFS	OR	60-day mortality
Bielza 2021 [21]	RCS	Spain	35.40%	Nursing home	NA	630	87	CFS	OR	30-day mortality
Mendes 2020 [23]	RCS	Switzerland	43%	Hospital	78.70%	235	86.3 (6.5)	CFS	HR	In-hospital mortality

*RCS* Retrospective cohort study, *PCS* prospective cohort study, *FNDQ* Frail Non-Disabled questionnaire, *HFRS* Hospital Frailty Risk Score, *HR* hazard ratio, *OR* odd ratio, *CFS* clinical frailty scale

### Meta-analysis of the effects of frailty on mortality

**Fig. 2:**
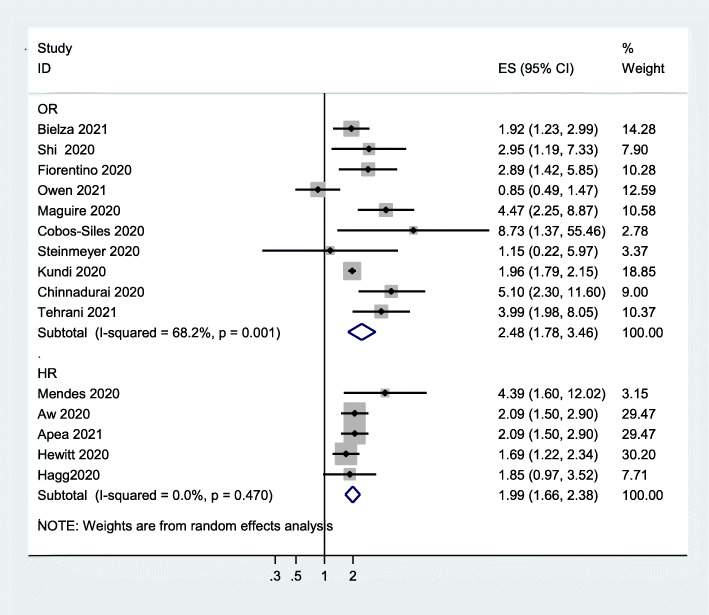
Meta-analysis of the effects of frailty on mortality among patients with COVID-19

Fifteen studies were included in the meta-analysis. Ten studies considered OR as the result of the association between frailty and mortality. The pooled OR value was 2.48 (95% CI: 1.78–3.46) among frail patients compared with COVID-19 patients without frailty. In addition, five studies used HR as an effect measure, with the pooled HR value of frail patients for mortality being 1.99 (95% CI: 1.66–2.38), both of which indicate that frailty can be an independent predictor for mortality among patients with COVID-19 (Fig. 2).

### Subgroup analysis was based on different populations

**Fig. 3:**
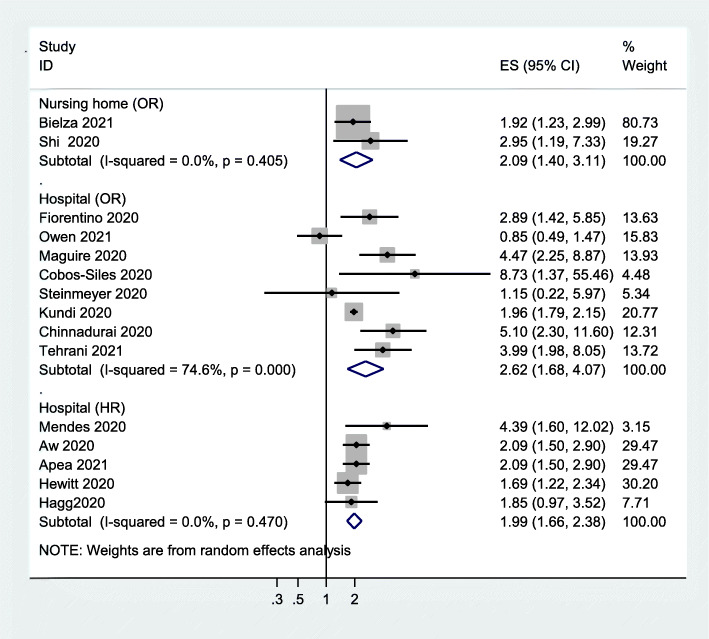
Meta-analysis of the effects of frailty on mortality based on different settings

A majority of studies focused on hospitalized patients, with two studies reported among nursing home residents. Older nursing home residents infected with COVID-19 and with frailty had a 2.09-fold risk of morality compared to nonfrail patients (assessed in 2 studies, pooled OR = 2.09, 95% CI: 1.40–3.11). Meanwhile, hospitalized patients also had similar results regardless of which effect measures were considered (assessed in 5 studies, pooled HR = 1.99, 95% CI: 1.66–2.38; assessed in 8 studies, pooled OR = 2.62, 95% CI: 1.68–4.07) (Fig. 3).

### Subgroup analysis was based on different frailty assessment scales

**Fig. 4:**
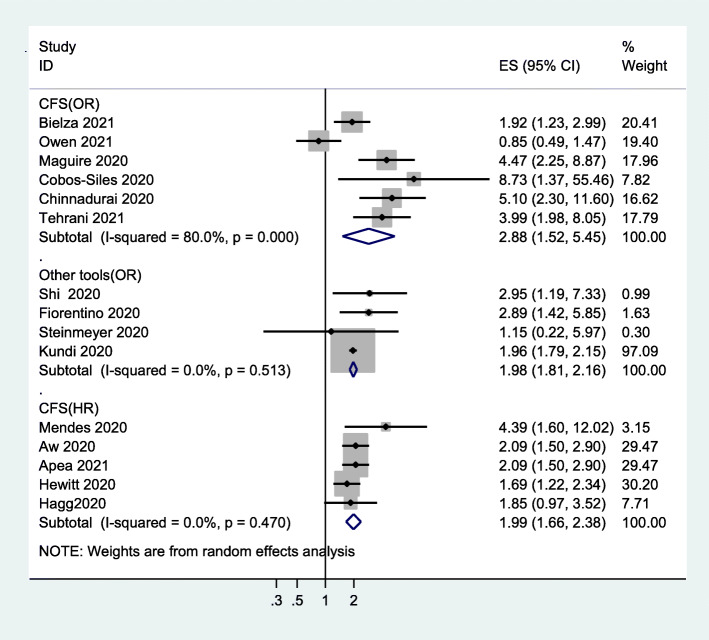
Meta-analysis of the effects of frailty on mortality based on different frailty assessment scales

We performed a subgroup analysis of the frailty assessment tool by considering CFS versus other tools. The results showed that the pooled HR and OR of frailty among patients who died, compared to those without frailty, were 1.99 (95% CI: 1.66–2.38) and 2.88 (95% CI: 1.52–5.45), respectively, when using CFS in 11 studies. Other frailty assessment instruments in four studies included the frailty index, hospital frailty risk score, frail nondisabled questionnaire, and palliative performance scale, with a pooled OR of 1.98 (95% CI: 1.81–2.16), as shown in Fig. 4.

### Subgroup analysis based on study design, geographic region, and different adjustment models

Seven studies were prospective cohort studies, and the others were retrospective cohort studies; thus, we performed a subgroup analysis based on study design. The results indicated a statistically significant association between frailty and mortality among cohort studies (assessed in 3 studies, pooled OR = 3.12, 95% CI: 1.60–6.09; assessed in 4 studies, pooled HR = 1.94, 95% CI: 1.62–2.32). Similar results were shown in the retrospective cohort study (assessed in 7 studies, pooled OR = 2.28, 95% CI: 1.34–3.88), as shown in Supplemental Fig. 2. We also performed a subgroup analysis based on geographic region due to the different prevalence levels of COVID-19 worldwide. The results found that the associations were higher in the USA (2 studies) than in European countries (13 studies), with both suggesting that patients with frailty have an incrementally greater risk of mortality than nonfrail COVID-19 patients (Supplemental Fig. 3). In addition, subgroup analysis based on the adjusted model showed that the association between frailty and mortality still existed in both models (5 unadjusted studies versus 10 adjusted studies) (Supplemental Fig. 4).

### Quality assessment

A majority of studies had used more than a seven-point score, and one study had six points according to the criterion of the Newcastle–Ottawa Scale (Table 2).

**Table 2:** Result of the Newcastle-Ottawa scale quality assessment

Author/years	Selection (4)				Comparability (2)	Outcome (3)			Quality (9)
	Representativeness of the exposed cohort	Selection of the non-exposed cohort	Ascertainment of exposure	Demonstration that outcome of interest was not present at start of study	Comparability of cohorts on the basis of the design or analysis	Assessment of outcome	Was follow-up long enough for outcome to occur	Adequacy of follow up of cohorts	
Kundi 2020 [18]	1	1	1	1	2	1	1	1	9
Hägg 2020 [12]	1	1	1	1	2	1	0	1	8
Aw 2020 [8]	1	1	1	1	2	1	0	1	8
Hewitt 2020 [17]	1	1	1	1	2	1	1	1	9
Shi 2020 [24]	1	1	1	1	2	1	0	1	8
Maguire 2020 [13]	1	1	1	1	1	1	0	1	7
Cobos-Siles 2020 [25]	1	1	1	1	1	1	0	1	7
Steinmeyer 2020 [16]	1	1	1	1	0	1	0	1	6
Owen 2021 [15]	1	1	1	1	2	1	0	1	8
Fiorentino 2020 [11]	1	1	1	1	1	1	0	1	7
Chinnadurai 2020 [19]	1	1	1	1	2	1	0	1	8
Apea 2021 [22]	1	1	1	1	1	1	0	1	7
Bielza 2021 [21]	1	1	1	1	1	1	0	1	7
Mendes 2020 [23]	1	1	1	1	1	1	0	1	7
Tehrani 2021 [20]	1	1	1	1	1	1	0	1	7

### Sensitivity analysis and potential publication bias

Begg’s test was used to determine whether there was a publication bias, and the results showed no potential bias (*p* = 0.147) (Supplemental Fig. 5). We also conducted a leave-one-out sensitivity analysis, and the results indicated that our study was stable and robust (Supplemental Fig. 6).

## Discussion

In this study, we found that COVID-19 patients with frailty have an increased risk of mortality than those without frailty, independent of study design, country, and setting, indicating that frailty could be a prognostic factor for clinicians to predict mortality and supporting the use of a frailty assessment to stratify high-risk hospitalized patients to provide appropriate medical care. This is the first meta-analysis with a large sample size, to the best of our knowledge, to explore the association between frailty and mortality among patients with COVID-19. Given the ongoing COVID-19 pandemic, the increasing number of deceased patients and the overwhelmed health care system, frailty screening could help clinicians establish a comprehensive prognostic tool for predicting mortality in patients with COVID-19 and early intervention for improving frailty syndrome to reduce mortality rates.

A large number of studies pooled the prevalence of frailty among different populations that resided in nursing homes [26] or communities [27], with prevalences of 52.3% (95% CI: 37.9–66.5%) and 17.4% (95% CI 14.4–20.7%), respectively. Our study found that the prevalence of frailty in patients with COVID-19 was similar to that in nursing home residents, but for both groups, it was higher than that for community-dwelling older adults. This is not an exceptional finding because the median or average age in these included studies was more than 70 years old. Increasing age was a risk factor for increased prevalence of frailty [28]. The oldest people (> 70 years old) were reported to be the most vulnerable population for SARS-CoV-2 infection, especially older residents of nursing homes. In addition, different frailty assessment tools and comorbidities were also factors that produced variations in prevalence.

Frailty as a predictor of mortality has widely been applied in different populations: community-dwelling older adults [29], nursing home residents [30], critically ill patients [31] and oncology patients [32], with HR values ranging from 1.8 to 3.39. A large number of evidence-based systematic reviews and meta-analyses found that frailty could be a predictive factor for adverse outcomes, including mortality [30], hospitalization [33], and readmission [34], which means that screening for frailty is very important in a clinical setting.

Although the mechanism between frailty and mortality has been described by previous studies, this association has not been completely explained because of the involvement of multiple complicated factors. Several reasons may account for this. First, compared to patients without frailty, patients with frailty suffer from a more vulnerable condition characterized by various observable deficits, such as a reduced physiologic reserve, chronic undernutrition and cognitive impairment, increasing the likelihood of an adverse outcome when patients are exposed to major negative stressors, including COVID-19 or surgery operations. Second, frailty involving the process of complex chronic inflammation and proinflammatory cytokines, such as C-reactive protein, tumor necrosis factor (TNF)-a, interleukin (IL) or interleukin-6, exacerbates the risk of mortality when patients contract COVID-19 [35]. A previous study reported that proinflammatory cytokines were enormously aggravated in patients with COVID-19 [36]. Proinflammatory cytokines related to frailty and COVID-19 cause an inflammatory storm in COVID-19 patients, progressing to the development of lung injury and later ARDS, intensifying the risk of mortality [14]. Third, older adults infected with SARS-CoV-2 have a high probability of developing severe status, requiring intensive medical care such as invasive ventilation, more drugs, and even extracorporeal circulation support. Frail older people are often unable to endure these invasive treatments or medical side effects, resulting in a greater likelihood of death during treatment. A previous meta-analysis showed that critically ill patients with frailty have a 1.71-fold risk of mortality. Thus, patients with both frailty and COVID-19 can develop a vicious cycle of impairment [31].

Our subgroup analysis, based on the frailty assessment tool, showed that frailty can be an independent predictor of mortality risk when using the clinical frailty scale and other frailty measurement tools. To date, several frailty measurement tools have been applied in different settings with various merits and demerits [37]. Optimal screening frailty scores should be practical, sensitive and available. Given the human-to-human transmission of COVID-19, being simple, less time-consuming, and accurate were the key points when clinicians considered using frailty instruments, especially for patients with a critical illness whose care requires more energy and time. CFS is considered the most common and efficient frailty assessment tool for a clinical setting because there are only five patient domains that need to be assessed [38]: basic ADLs, instrumental ADLs, chronic medical conditions that require drugs, exercise, and appearing fitter compared with patients of similar age. However, other tools need to be evaluated on many different aspects, such as the frailty index, which includes 35 items [39]. Previous studies have validated CFS as a predictor of adverse outcomes among hospitalized patients [40, 41]. Additionally, our study also confirmed that frail patients assessed by CFS have an increased risk of morality compared to those without frailty. Recently, the National Institute for Clinical Excellence (NICE) published a guideline that recommends the CFS as an assessment tool to evaluate frailty in patients with COVID-19 [42]. Other subgroup analyses based on different designs and countries also showed similar results, meaning that the association between frailty and mortality in patients with COVID-19 is reliable and stable.

Our subgroup analysis shows that the association between frailty and mortality exists in different settings, both in hospitalized patients and nursing home residents. It is estimated that 2 in 5 US deaths from COVID-19 occurred in long-term care facilities or nursing homes. The main reason why nursing home residents are the most vulnerable group for COVID-19 is that older residents often suffer from multimorbidity, such as heart disease, diabetes, and kidney disease, overlapping with frailty, generating a vicious cycle, which was reported to be a risk of mortality [43, 44]. In fact, COVID-19 patients need to be treated at the designed hospital first in case of contacting other non-COVID-19 patients. Given the surging number of COVID-19 patients in the USA and Europe, hospitals are overwhelmed by COVID-19 patients, and medical staff are under great pressure. Government authorities and policymakers require most nursing home residents to remain in their facility. However, preventing COVID-19 transmission in nursing homes is very challenging but important.

Our systematic review and meta-analysis has some strengths and limitations. To the best of our knowledge, this is the first systematic review and meta-analysis study, which included 23,944 participants, to explore the association between frailty and mortality in patients with COVID-19 using comprehensive analysis methods. Our study may help answer the question of whether frailty could be a stratified tool for COVID-19 patients, and our results indicate that frailty is an independent predictor of mortality. However, there are also some limitations, and we need to remain cautious about the conclusions. First, two studies included nursing home residents, requiring more studies to confirm the impact of frailty on mortality in nursing home residents to guide policymakers to better manage this valued, high-risk group. Second, the numbers for some important frailty assessment tools, such as the frailty index or HFR, were limited, influencing the subgroup analysis results based on the frailty assessment tool. Five studies did not provide the adjusted model for the HR and OR values of frailty on mortality; therefore, the pooled HR and OR might be an overestimate. However, we performed a sensitivity analysis based on unadjustment and adjustment models and found that the association between frailty and mortality existed in both models. Third, we excluded some important studies that considered the frailty score as a continuous variable, which may have led some relevant information to be missed.

## Conclusion

This systematic review and meta-analysis, which summarizes the evidence of the impact of frailty on mortality in COVID-19 patients, shows that COVID-19 patients with frailty have an increased risk of mortality compared with nonfrail patients with COVID-19, and this association is independent of geographic region, study design and setting. Overall, the assessment of frailty can help clinicians stratify the category risk of older patients with COVID-19 to help clinical healthcare workers manage and balance the benefits and risk for patients. Thus, multidimensional and effective medical care or intervention are required for this group, with the aim of reducing mortality rates.

## Supplementary Information

**Additional file 1: Supplemental Figure 1.** Pooled prevalence of frailty among patients with COVID-19.

**Additional file 2: Supplemental Figure 2.** Subgroup meta-analysis of the effects of frailty on mortality based on study design.

**Additional file 3: Supplemental Figure 3.** Subgroup meta-analysis of the effects of frailty on mortality based on geographic region.

**Additional file 4: Supplemental Figure 4.** Subgroup meta-analysis of the effects of frailty on mortality according to different models.

**Additional file 5: Supplemental Figure 5.** Begg’s test for publication bias.

**Additional file 6: Supplemental Figure 6.** Sensitivity analysis of the association between frailty and mortality.

**Additional file 7: Supplemental Table S1.**

**Additional file 8: Supplemental Table S2.**

**Additional file 9.** Supplemental file 1.

## Data Availability

The datasets generated and/or analysed during the current study are available in the PubMed database.

## Abbreviations

COVID-19: Coronavirus disease 2019
CFS: clinical frailty scale
NOS: Newcastle–Ottawa Scale
HR: hazard ratio
CI: confidence interval
OR: odds ratio
