# Supplementary material for: Frailty as a predictor of mortality among patients with COVID-19: a systematic review and meta-analysis
Source: BMC Geriatr. 2021 Mar 17;21:186. doi: 10.1186/s12877-021-02138-5 (PMC7968577; doi:10.1186/s12877-021-02138-5)
Supplement: Supplementary file 7 — Additional file 7: Supplemental Table S1. [file 12877_2021_2138_MOESM7_ESM.docx]

**Supplemental Table1：Adjustment variables for each study.**

| **Author** | **Adjustment** |
| --- | --- |
| Kundi 2020 | Age, sex, comorbidities |
| Hägg 2020 | Age, gender, Hospital Frailty Risk Score, charlson comorbidity index, AKI, Harrell C |
| Aw 2020 | Age, sex, National Early Warning Score, Index of Multiple deprivation quintile, hospital spells, ethnicity |
| Hewitt 2020 | Age group, sex, smoking, C-reactive protein, diabetes, coronary artery disease, hypertension, renal  function |
| Shi 2020 | Unadjusted |
| Maguire 2020 | Unadjusted |
| Cobos-Siles 2020 | Age |
| Steinmeyer  2020 | Unadjusted |
| Owen 2021 | Unadjusted |
| Fiorentino 2020 | Age, ethnicity, gender, BMI, charlson comorbidity index, Do not intubate order,dialysis, insurance status, |
| Chinnadurai 2020 | Age, care home resident, frailty, smoking,  BMI, CVD, respiratory diseases, neutrophil: lymphocyte ratio, C-reactive protein (CRP), eGFR, and acute kidney injury |
| Apea 2021 | Age, sex |
| Tehrani 2021 | Unadjusted |
| Bielza 2021 | Age, Sex, hospital admission, dementia, COPD, hypertension, sleep apnea syndrome, dyspnea, epileptic seizures, abdominal pain, cough, anosmia, sever case |
| Mendes 2020 | Sex |
