# Supplementary material for: Frailty as a predictor of mortality among patients with COVID-19: a systematic review and meta-analysis
Source: BMC Geriatr. 2021 Mar 17;21:186. doi: 10.1186/s12877-021-02138-5 (PMC7968577; doi:10.1186/s12877-021-02138-5)
Supplement: Supplementary file 9 — Additional file 9. Supplemental file 1. [file 12877_2021_2138_MOESM9_ESM.docx]

**Supplement files Search strategy**

**PubMed**

#1 Coronavirus* OR “Coronavirus Infection” OR “COVID-19” OR “Coronavirus Infection Disease 2019” OR “2019 Novel Coronavirus Infection” OR “2019-nCoV Infection” OR “2019 nCoV Infection” OR “2019-nCoV Infections” OR “SARS-CoV-2” OR “Novel Coronavirus Pneumonia” OR “2019 novel coronavirus” OR “coronavirus disease 2019” OR “nCoV” OR COVID*)

#2 frail*

#3 mortality or mortality [Mesh] or death or "Death"[Mesh] or survival or "Survival"[Mesh]

#4 #1 and #2 and #3
