# Supplementary material for: Frailty as a predictor of mortality among patients with COVID-19: a systematic review and meta-analysis
Source: BMC Geriatr. 2021 Mar 17;21:186. doi: 10.1186/s12877-021-02138-5 (PMC7968577; doi:10.1186/s12877-021-02138-5)
Supplement: Supplementary file 7 [file 12877_2021_2138_MOESM4_ESM.docx]

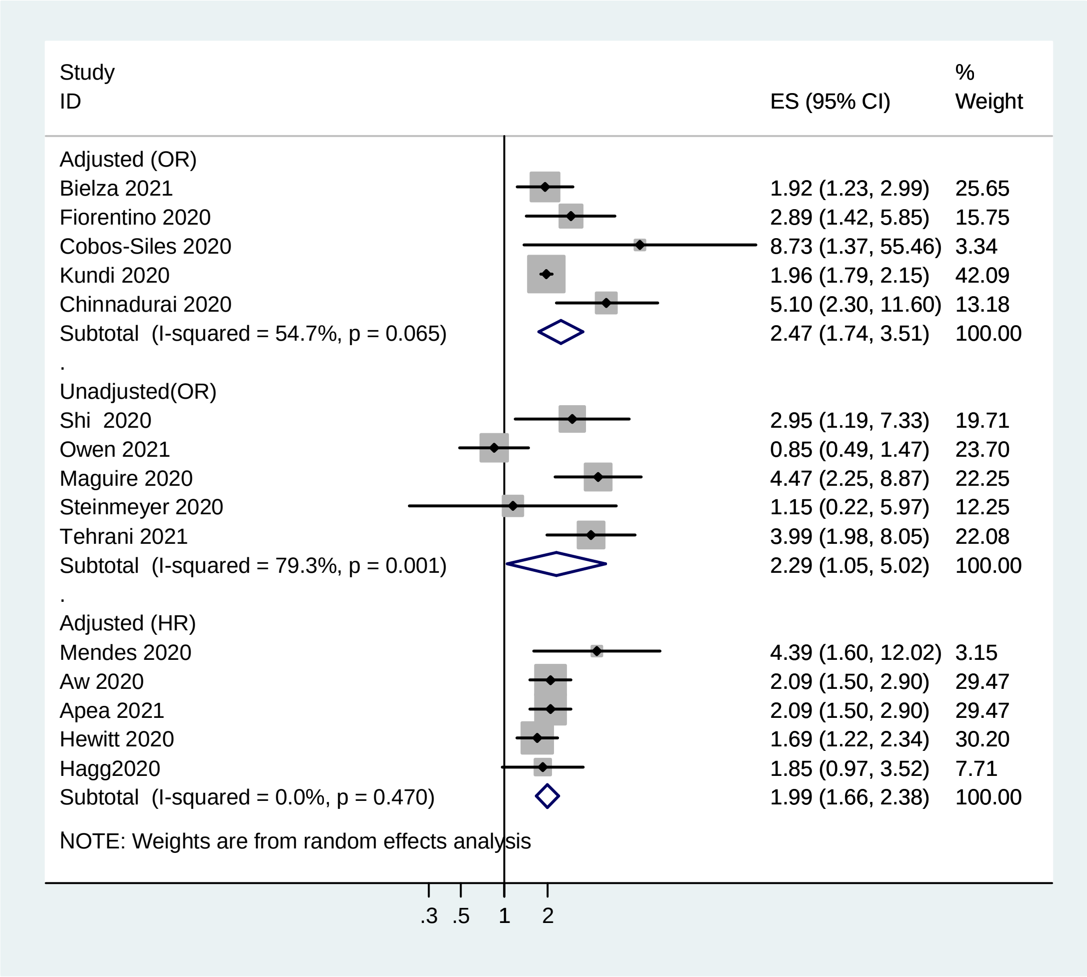
Study
ID
ES (95% CI)
%
Weight
Adjusted (OR)
Bielza 2021 1.92 (1.23, 2.99) 25.65
Fiorentino 2020 2.89 (1.42, 5.85) 15.75
Cobos-Siles 2020 8.73 (1.37, 55.46) 3.34
Kundi 2020 1.96 (1.79, 2.15) 42.09
Chinnadurai 2020 5.10 (2.30, 11.60) 13.18
Subtotal (I-squared = 54.7%, p = 0.065) 2.47 (1.74, 3.51) 100.00
.
Unadjusted(OR)
Shi 2020 2.95 (1.19, 7.33) 19.71
Owen 2021 0.85 (0.49, 1.47) 23.70
Maguire 2020 4.47 (2.25, 8.87) 22.25
Steinmeyer 2020 1.15 (0.22, 5.97) 12.25
Tehrani 2021 3.99 (1.98, 8.05) 22.08
Subtotal (I-squared = 79.3%, p = 0.001) 2.29 (1.05, 5.02) 100.00
.
Adjusted (HR)
Mendes 2020 4.39 (1.60, 12.02) 3.15
Aw 2020 2.09 (1.50, 2.90) 29.47
Apea 2021 2.09 (1.50, 2.90) 29.47
Hewitt 2020 1.69 (1.22, 2.34) 30.20
Hagg2020 1.85 (0.97, 3.52) 7.71
Subtotal (I-squared = 0.0%, p = 0.470) 1.99 (1.66, 2.38) 100.00
NOTE: Weights are from random effects analysis
.3
.5
1
2
