# Supplementary material for: Frailty as a predictor of mortality among patients with COVID-19: a systematic review and meta-analysis
Source: BMC Geriatr. 2021 Mar 17;21:186. doi: 10.1186/s12877-021-02138-5 (PMC7968577; doi:10.1186/s12877-021-02138-5)
Supplement: Supplementary file 8 [file 12877_2021_2138_MOESM5_ESM.docx]

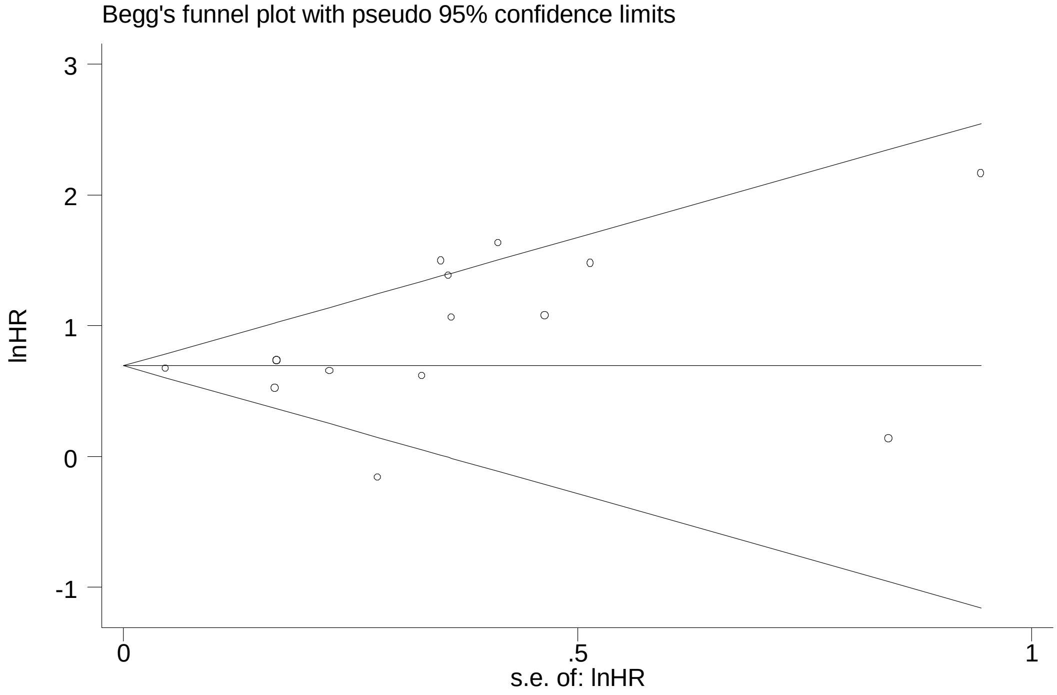
Begg's funnel plot with pseudo 95% confidence limits
3
2
1
0
-1
lnHR
0
.5
1
s.e. of: lnHR
