# Supplementary material for: Frailty as a predictor of mortality among patients with COVID-19: a systematic review and meta-analysis
Source: BMC Geriatr. 2021 Mar 17;21:186. doi: 10.1186/s12877-021-02138-5 (PMC7968577; doi:10.1186/s12877-021-02138-5)
Supplement: Supplementary file 9 [file 12877_2021_2138_MOESM6_ESM.docx]

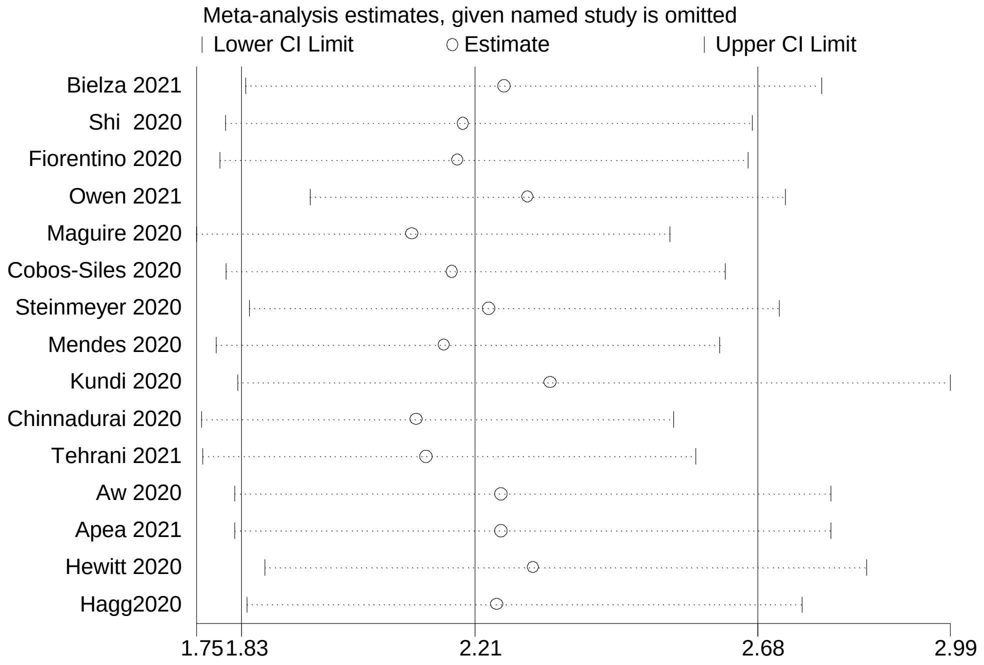
Meta-analysis estimates, given named study is omitted
Lower CI Limit
Estimate
Upper CI Limit
Bielza 2021
Shi 2020
Fiorentino 2020
Owen 2021
Maguire 2020
Cobos-Siles 2020
Steinmeyer 2020
Mendes 2020
Kundi 2020
Chinnadurai 2020
Tehrani 2021
Aw 2020
Apea 2021
Hewitt 2020
Hagg2020
1.75
1.83
2.21
2.68
2.99
